# Benzine Poisoning

**Published:** 1895-07-06

**Authors:** 


### THERAPEUTIC NOTES.

### BENZINE POISONING.

Dr. Ernest Rosenthal (Cbl. f. innere Med., 1894) reports a case of benzine poisoning in which a child, eighteen months old, drank a teaspoonful. Fifteen minutes later it was in a stupefied condition, and the stomach was promptly emptied, some benzine with blood-stained mucus and particles of milk coming away. A good deal had evidently been absorbed, and in about an hour the symptoms had reached their height, the pulse being scarcely perceptible, respiration more frequent, and the child narcotised. Six hours later it was still slightly stupefied, weak, and staggering, and the respiration somewhat too fast. There was no albumin or sugar in the urine. It recovered perfectly. Rosenthal states that he had a patient who took benzine habitually as a substitute for alcohol, to which he had been formerly much addicted. He preferred the stupefying effects of the benzine.

